# Is it recorded in the notes? Documentation of end-of-life care and preferred place to die discussions in the final weeks of life

**DOI:** 10.1186/1472-684X-10-18

**Published:** 2011-11-04

**Authors:** Karen Cox, Nima Moghaddam, Kathryn Almack, Kristian Pollock, Jane Seymour

**Affiliations:** 1 The Sue Ryder Care Centre for the study of supportive, palliative and end of life care, School of Nursing, Midwifery and Physiotherapy, University of Nottingham, Queen's Medical Centre, Nottingham, NG7 2UH, UK; 2 University of Lincoln, Faculty of Health, Life and Social Sciences, 1st Floor, Bridge House Brayford Pool, Lincoln, LN6 7TS, UK

## Abstract

**Background:**

Over the past ten years there has been an increasing focus on the need for improving the experience of end of life care. A number of policy initiatives have been introduced to develop approaches to discussing and documenting individual preferences for end of life care, in particular preferred place to die.

**Methods:**

The aim was to investigate practice in relation to discussing and documenting end of life care and preferred place to die in the last 4 weeks of life with patients and their families. The study utilised an audit of 65 case notes, alongside four group interviews with a mix of health care professionals involved in palliative care provision.

**Results:**

While there was evidence that discussions relating to end of life care and preferred place to die had taken place in around half of the audited case notes, there appeared to be a lack of a systematic approach to the recording of discussions with patients or carers about these kind of issues. Health care staff subsequently highlighted that initiating discussions about end of life care and preferences in relation to place of death was challenging and that the recording and tracking of such preferences was problematic.

**Conclusions:**

Further work is required to establish how information may be adequately recorded, revised and transferred across services to ensure that patients' preferences in relation to end of life care and place of death are, as far as possible, achieved.

## Background

Debates relating to what makes a 'good death' often place particular emphasis on being pain free, peaceful and dignified but without dying being over-prolonged [1-3]. One additional element being highlighted as increasingly important to the concept of a 'good death' is identifying patients' preferences for how wish to be cared for at the end of their lives and where they wish to die [4,5].

Realising patients' preferences for the kind of care they wish to receive and the place of their death is often taken as a key indicator of quality of end of life care and there is evidence that if patients' preferences are discussed and documented they are more likely to be realised [6]. In the United Kingdom (UK) there are increasing calls from policy makers, and some healthcare professionals for greater openness in the way that death and dying is talked about throughout society [4,5]. Routine discussion of patient and carer preferences for end of life care is advocated as a means of promoting such openness and also as an effective lever for improving the experience of end of life care [7]. Building on the work of the National End of Life Care programme launched in 2004, The National End of Life Care Strategy for England [5] includes explicit reference to the need to increase public awareness of death and dying through discussing individual preferences for end of life care and notes specifically that *'All people approaching the end of life need to have their needs assessed, their wishes and preferences discussed and an agreed set of actions reflecting the choices they make about their care recorded in a care plan' *(p6, para 14).

A number of initiatives have been developed and evaluated that have specifically encouraged the discussion and documentation of preferences for care at the end of life. These include the National End of Life Care Programme launched in 2004 and the National Institute for Clinical Excellence (NICE) guidance on Improving Supportive and Palliative Care for Adults with Cancer [7] which highlighted that many individuals with cancer had poorly co-ordinated care and little choice about where they die. The guidance specifies the use of tools such as the Gold Standards Framework [8], Preferred Place of Care Plan [9] and the Liverpool Care Pathway [10] as appropriate mechanisms for supporting the provision of quality care at the end of life. Identifying that patients were under the care of providers working within these guidelines or on a care pathway that considered these issues was deemed to be beneficial to the quality of care they subsequently received. More recent guidelines from the Department of Health [5], the Advance Care Planning Guidelines produced in 2009 by the Royal College of Physicians [11] and the draft guidance on Quality Standards in End of Life Care currently being developed by NIHCE [12] provide further support for the requirement to discuss and record preferences for care and place of death with patients and their families as they approach the end of their lives. There is some evidence that the use of care planning tools can result in improved documentation and that this can improve the decision making process in end of life care. For example, a randomised controlled trial of advance care planning among hospitalized patients aged over 80 found that it improved end of life care and patient and family satisfaction and reduced stress, anxiety, and depression in surviving relatives [6], in addition, an audit of the care provided to patients who died in UK hospitals within 96 hours of admission found better end of life care (in terms of access to palliative care advice and communication with relatives and between health care teams) to be associated with care planning tools such as the Liverpool Care Pathway [13].

The initiatives outlined above have led to an increased focus on the need to prompt discussion and record patients' preferences for care when they approach the end of their lives. However, there is evidence that engaging in care planning of this nature is not straightforward for either health care professionals or patients. The process of eliciting and documenting preferences for care at the end of life has been subject to some scrutiny over recent years and in a number of settings [14-16]. These studies have demonstrated that, in spite of the plethora of initiatives and tools available to support discussion and record keeping, staff can find it difficult to initiate conversations about death and dying and then make a meaningful record of them. Even when conversations do take place patient preferences can change over time making the recording and tracking of decisions challenging [15]. A retrospective review of the medical records of 310 adults who died in a Western Canadian Region [17] identified that while there were clear institutional policies in place these were not always followed in practice in terms of documentation of discussions with patients and families [17]. It is important to know, therefore, what is happening in practice in relation to professional engagement in discussing and planning care for end of life and subsequent documentation, as it serves to highlight where further initiatives may be required in order to support best practice in this area.

The findings presented in this paper are taken from a larger piece of work which sought to examine palliative and end of life care delivery in both cancer and non cancer (heart failure) patient populations. The element of the project reported in this paper had the specific objective of auditing the recording of discussions relating to end of life care, in particular preferences and outcomes for place of care while dying. Data were also collected that related to the presence/absence of Do Not Resuscitate (DNR) orders and whether an individual was identified as being on a particular care pathway related to end of life care.

## Methods

The study design incorporated an audit of case notes on a sample of deaths occurring over a 12 month period and complementary group interviews with healthcare professionals. Four study sites were selected from across a Regional Cancer Network for inclusion in this element of the work. The choice of sites was discussed with the project steering group and ensured palliative care provision across both cancer and non-cancer populations and allowed for comparison across organisational boundaries. Study sites included: a Hospital Specialist Palliative Care (SPC) service, a GP practice service using the Gold Standards Framework, a Heart Failure Community Matron service (home based nursing service for people with heart failure living in the community) and a Nursing Care home. Two sampling fractions were defined for the purposes of selecting deaths from services. All but one care service, the Hospital Specialist Palliative Care (SPC) service had a similar number of deaths over the specified period, and a sampling fraction of 50% was used. The SPC service had a much larger number of deaths over the specified period; consequently a decision was made to sample a smaller (5%) fraction of cases from this population. For all services, a true random number generator was used to select cases. Table 1 shows the number of deaths/sampled cases for each service. Due to limitations of time, resources and approvals, the researchers sampled from records that were kept by and within the services that they had access to and focussed on care documented in the last four weeks of life.

**Table 1:** Sampling by service

Service	Number of deaths (Jan 05 - Dec 06)	Sampling fraction	Number of sampled deaths/cases (random selection)
GSF GP Practice	38	50%	19
Heart Failure Community Matrons	30	50%	15
Hospital Specialist Palliative care service	307*	5%	15
Non-GSF Nursing Care Home	32	50%	16
			
*TOTAL*	*407*		*65*

*This service was based in a hospital but provided hospice care support as well. The focus here was on the hospital element and this figure represents the number of deaths in hospital only.

**Figure 1:**
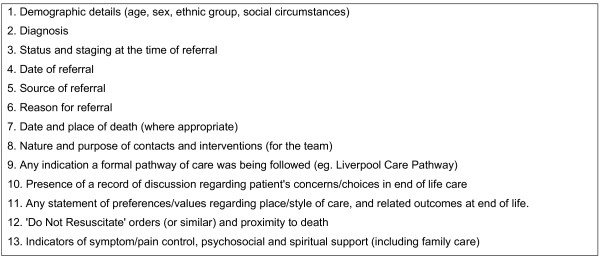
**Information collected from patient case notes**.

A service audit tool was developed to be sensitive to the aims of the End of Life Care Programme (http://www.endoflifecareforadults.nhs.uk/), and was refined through consultation with the project steering group. The full details of the categories of data extracted from the sampled case notes using the audit tool is listed in Figure 1. Records maintained by/within case services were examined by two researchers (KA, NM) to ensure a consistent approach to data extraction. Information extracted from the notes was transferred to the audit document and recorded in either a tick box format (eg. indicating the presence or absence of a DNR decision, the presence or absence of end of life care discussions), short written statements (eg. Place of care in week 1, 2, 3, 4) or a verbatim record of the written evidence (eg. of care preference discussions with patients or family members). While observation of practice may have been revealing, at this point in the study we wanted to establish practice that had taken place already in terms of what was actually documented.

Group interviews were conducted with health care professionals associated with each of the study sites who were responsible for the provision of palliative care services. An aide-memoire of topics was used to guide these interview conversations. Topics covered included: views on end of life care policy; how discussions about preferred place of care and death are initiated with patients and their families; how these conversations (and decisions) are documented and communicated; and how physical and psychosocial needs are assessed and met.

Interviews in a group setting are particularly useful when it is important to gain data from a group where interactions between members can enhance the data collected as they discuss and reflect on each other's responses [18]. It was intended that group interview data would serve in part to contextualise findings from the audit: elucidating approaches to record-keeping and identifying disparities between reported and recorded practice. Four group interviews were conducted in total consisting of between 2-4 participants with a total of 13 individuals taking part. Participants included a GP, two District Nurses, a Practice Manager, two Community Matrons, two Macmillan Nurses, a Specialist Palliative Care Team Manager, a Nursing Home Manager, a Care Co-ordinator and two Registered Nurses.

Ethical approval was obtained from the Local Research Ethics Committee (ref no. 06/Q2404/123) and relevant NHS Trust approvals were also secured.

### Data analysis

Data extracted from case notes during the audit were entered into SPSS v.17 for a basic descriptive analysis. Data were not suitable for comparative significance testing. The group interviews were recorded and transcribed and entered into the qualitative data support and analysis package NVIVO. All the transcripts were read by the group facilitator (KA or NM) and initially coded in detail using a constant comparative approach [19]. The initial set of codes was verified by the broader research team and developed and refined as more interviews were undertaken. These codes were subsequently grouped into broader categories of data which provided a description of the underlying meaning of the interview text.

## Results

Of the 65 cases sampled, 22 were female, 43 male, with a mean age of 76 yrs (range 56-99), 36 had a malignancy diagnosed, 24 had a non malignant diagnosis (heart failure) and 5 had no diagnosis recorded. Findings of the audit are organised into two sections below: (1) end-of-life discussions and (2) preferences and outcomes for place of death. These are followed by (3) findings from the interviews.

### (1) End-of-life discussions (see table 2)

#### Discussions with patients about end of life care

End of life care discussions with patients were documented for the majority of cases sampled from the GP Practice (15 out of 19) and Hospital SPC Service (11 out of 15) both cancer-focused services. There were recorded discussions for a third of patients (5 out of 15) sampled from the Heart Failure Community Matron caseload. There were no records of patient discussions about EOL care or concerns in the Care Home sample. Recorded discussions covered a range of topics, these included: what palliative care was; the need for psychological support; preferred place of care; preferred place to die; desire for information about euthanasia. Records of discussions were however variable in terms of detail, ranging from minimal:

**Table 2:** Discussions relating to end of life care recorded in the last four weeks of life

	GSF GP Practice		Heart Failure Community Matrons		Hospital Specialist Palliative Care Service		Non-GSF Nursing Care Home		Total	
	***n***	***(%)***	***n***	***(%)***	***n***	***(%)***	***n***	***(%)***	***n***	***(%)***
*Evidence of discussion about EOL issues with patient?*										
Yes	15	(79)	5	(33)	11	(73)			31	(48)
No	4	(21)	10	(67)	4	(27)	16	(100)	34	(52)
										
*Evidence of discussion about EOL issues with carer?*										
Yes	7	(37)	6	(40)	11	(73)	11	(69)	35	(54)
No	12	(63)	9	(60)	4	(27)	5	(31)	30	(46)
										
*Evidence of a DNR order?*										
Yes					8	(53)	6	(38)	14	(22)
No	19	(100)	15	(100)	7	(47)	10	(63)	51	(79)
										
*Recognised care pathway recorded?*										
Yes	1	(5)					5	(31)	6	(9)
No	18	(95)	15	(100)	15	(100)	11	(69)	59	(91)

Percentages may not add up to 100 due to rounding.

Cancer diagnosis discussed

To more detailed accounts:

*Discussion with doctor at home visit. Philosophical about dying, not afraid but sorry to leave family behind. Preferred place of care is home. Good family support, daughter in village.......Patient keen to stay at home to end his days but keen to come into (hospice) to have a bit of a break and help with symptoms. Knows he is dying and 'has had enough'*.

#### Discussions with carers about end of life care

Discussions with a family carer were documented for the majority of cases sampled from the Hospital SPC Service (11 out of 15) and the Nursing Care Home (11 out of 16). There were recorded discussions with just over a third of carers in cases sampled from the GSF GP practice. Six out of 15 case records sampled from the Heart Failure Community Matron caseload contained evidence of carer discussions.

Recorded discussions covered a range of topics. These included: care pathways; awareness of diagnosis; concerns about eating; power of attorney; preferences for place of care; resuscitation; likelihood of imminent death; patients' symptoms; burden of caring responsibility. These records were also variable in terms of detail, ranging from minimal:

*Dr discussed Liverpool care pathway- both sons agreed*.

To more detailed accounts:

*Medical care practitioner from X advised admission into hospital. Care home staff contacted family. Wife declined admission, confident that if her husband was going to die he would be well looked after in the care home and that she was happy for him to die there*.

#### Do Not Resuscitate (DNR) orders

There was no evidence of DNR orders in case records sampled from the GSF GP Practice or Heart Failure Community Matron services. Of the sampled Hospital SPC Service cases, just over half had recorded DNR orders (8 out of 15). DNR orders were evident for 6 of the 16 sampled Nursing Care Home cases.

#### Recognised EOL care pathways

The Nursing Care Home documented Liverpool Care Pathway (LCP) usage for almost one third (5 out of 16) of sampled patients. LCP use was also documented for 1 of 19 patients sampled from the GSF GP Practice. There was no documentary evidence of pathway use in the other care services.

### 2) Preferences and Outcomes in relation to place of death (see table 3)

#### Preferred place to die

Preferred place to die was explicitly recorded for the majority of Hospital SPC cases (11 out of 15) and for around half of the GSF GP Practice (10 out of 19) and Nursing Care Home cases (8 out of 16). Only three out of 15 cases from the HF Community Matrons service had a recorded preferred place to die (in the last four weeks of life). Where recorded, the patient's current residence (own home or care home) was most frequently specified as the preferred place to die. There were notable exceptions, however. For cases where a hospital or hospice preference was specified, records indicated that these preferences had emerged in response to recent deterioration whereas preferences for home were sometimes more long-standing (pre-dating recent deterioration). In some cases, records did show changes in preferred place over time (within the last four weeks of life). Preferences were often recorded within accounts of discussions with carers.

**Table 3:** Preferences and outcomes in relation to place of death recorded in the last four weeks of life

	GSF GP Practice		Heart Failure Community Matrons		Hospital Specialist Palliative Care Service		Non-GSF Nursing Care Home		Total	
	***n***	***(%)***	***n***	***(%)***	***n***	***(%)***	***n***	***(%)***	***n***	***(%)***
*Evidence of recorded preferred place to die?*										
Yes	10	(53)	3	(100)	2	(18)	8	(100)	20	(49)
No	9	(47)			9	(82)			12	(51)
										
*Preferred Place to die (where recorded)*^a^										
Patient's own home	8	(80)	2	(67)	7	(64)			17	(53)
Hospital					2	(18)	1	(13)	3	(9)
Hospice	1	(10)							1	(3)
Care home	1	(10)	1	(33)	1	(9)	7	(88)	10	(31)
Patient-carer differences					1^b^	(9)			1	(3)
										
*Place of death (all cases)*										
Patient's own home	11	(58)	6	(40)					17	(26)
Hospital	5	(26)	5	(33)	15	(100)	4	(25)	29	(45)
Hospice	1	(5)							1	(2)
Care home	1	(5)	2	(13)			12	(75)	15	(23)
Not recorded	1	(5)	2	(13)					3	(5)
										
*Died in preferred place? (Where recorded)*										
Yes	7	(70)	3	(100)	2	(18)	8	(100)	20	(63)
No	3	(30)			9	(82)			12	(38)

Percentages may not add up to 100 due to rounding.

^a^Where multiple PPC statements were made, the final PPC is represented.

^b^Patient wanted to go home, carer wanted the patient to stay in hospital: discharge plans were suspended.

#### Meeting preferred place to die

Across services, the majority of cases for which preferred place to die was documented died in their preferred place (20 out of 32). Against this general observation, only a minority of cases sampled from the Hospital SPC Service died in their preferred place (2 out of 11).

### 3) Group interview findings

Group interviews revealed a number of issues relating to the recording of discussions about end of life concerns with patients and documenting preferences for place of death. Three major issues emerged from the analysis of the interview material, two relating primarily to the limitations associated with record keeping. The major issues included: lack of detail in documentation; the practice of verbally communicating information to colleagues rather than making written notes; and the ethical concerns raised by the perceived requirement to discuss and document choices. These three issues are discussed below. Where extracts from the group interviews are used they are referred to only by site. The individual who made the comment is not further identified to preserve anonymity.

#### Lack of detail

During group discussions it was noted that there were a number of challenges associated with documenting what could be detailed and lengthy discussions. Group interview participants identified that recording such discussions in any detail was difficult due both to a lack of time and also because the detail of these conversations could not be easily conveyed onto paper. Many group interview participants noted that patient records did not always reflect the reality of the conversations that had been undertaken, as noted in the following extracts:

*I think what we would record is if there was something absolutely specific that was likely to be other people going in ought to know that, if they've said something specific, or if there's something they're worrying about and you want to highlight that this is a worry, and this is what we've discussed. But a lot of it is not recorded*.

(Site one)

I: And then so having these conversations, what about recording those conversations, do you have a set way of documenting these kind of end of life concerns and recording preferences?

R: Well on the front of our referral sheet we actually have a space that says preferred place of care, preferred place of death; where they are, preferred place of care, preferred place of death, and actual date of death, but we'll write on there. So if I saw somebody today and then it said they wanted to stay in hospital, and then they changed their mind and wanted to die at home, you'd just alter it and actually date it, so that you know where you're up to. But you could also write it in the text of your note documentation anyway, Mrs X discussed where she wants to be at the end of her life, became distressed or whatever, and just put something about it. You don't go into the deep things that have been said, but you get the gist of what's being discussed. So that you know, you'd perhaps put daughter was with her, so that who knows and who doesn't know. (Site three)

Explanations for why there was a lack of recording of detail about conversations relating to end of life care indicated that health care staff were focussed on recording tasks and physical aspects of care rather than the psychosocial elements, for example:

And we're very, probably we are still, as nurses, fairly task orientated with our record keeping, so it's usually if something has been done or it needs recording, you probably found that in district nurses notes. If something's been done, it will have been recorded, but if it's general discussions about issues and supporting the patient at home, then it probably won't be recorded. (Site two)

But, at the same time, it's very difficult to actually do everything that you're supposed to do. And it's like which is more important delivering the care or writing about it? (Site four)

#### Carrying information in one's head

During the discussions in the group interviews participants went on to describe how they carried much of their information about patients 'in their heads' and communicated this verbally with colleagues rather than relying on the recorded notes to support transfer of information between staff and across organisational boundaries. This system of passing on information verbally was presented as a necessarily practical solution in view of the constraints of maintaining full records of conversations and decisions. For example:

Very often as well, even if it's not hugely documented, we will pass on actual statements of the conversation, to give a proper picture of how they're particularly feeling at that time, especially at the end stage, you know. (Site one)

However, interview participants identified that the practice of verbally communicating information to colleagues meant records don't adequately represent the actual service delivered and could also lead to problems if colleagues were off sick.

*There is a problem with covering each other's caseloads because obviously I think a lot of the care you deliver and the sort of way you go with that patient is actually in your own head because there's only so much you can document (Site two)*.

In particular one example was given where lack of detail in terms of documentation of an advance care decision could cause problems later in the care trajectory if carers' wishes were at odds with those of the patient.

The thing is, going back to your documentary evidence as well, because of the introduction of the Capacity Act, and the 'do not resuscitate', you've got to be very careful what you're documenting, because before we had an issue where the carer turned round and said, the patient said, I don't want to be resuscitated in any way, shape or form, but the carer said, if he was unconscious, I would phone for an ambulance, and then the ambulance would have to resuscitate. Now with the introduction of the Capacity Act, then that, we can actually override that, and we can, you know, we've got a lot more say of what's going to, the patient's got a lot more say in what's going to happen, rather than the carer. So you've got to be really specific in what you're actually recording. (Site one)

#### Ethical Concerns

Some participants noted how the move towards opening up such discussions and the resultant requirement to document discussions and decisions raised tensions for them. For example, the use of tools associated with various end of life care initiatives led to perceptions that healthcare staff *had *to ask questions about end of life care preferences such as where people would prefer to die, when in practice they felt reluctant to do so or judged the timing of such conversations to require more sensitivity than what they perceived the introduction of these tools allowed for.

It's quite interesting that is, because that's one of the big things I've noticed in being involved more clinically over the last year, is I've felt a real pressure to know where everybody wants to be cared for, to know where they want to die. And you have to say, 'Hold on a minute, let's just assess how the patient is, what's appropriate at this time, it's the first time I've met them, is it appropriate to start talking about where they want to die?' And I don't know about you, but I've certainly felt that, gosh, I need to get out of them where he wants to die, otherwise I'm failing in my role, which is quite interesting, that's a real change. (Site three)

While group interview participants were acutely aware of the importance of initiating conversations about end of life care and documenting outcomes they also recognised that on some occasions it was not appropriate to be overly prescriptive about this requirement or to follow blanket policy guidelines blindly. In many instances focus group participants reflected on how they tended to be guided by the individual patient in relation to initiating conversations about end of life care and wait for patients to give them cues, rather than taking the initiative over whether and when such conversations took place.

## Discussion

There appeared to be a lack of a systematic approach to the recording of discussions with patients or carers about end of life care issues. Even when discussions were recorded researchers auditing the notes and those interviewed identified there was substantial variability in terms of the content, depth and detail of the discussion. Lack of detail is in and of itself not necessarily a problem particularly if any decision made is clearly recorded for example a preference about where one would like to die. However, lack of detail and contextual information as to how and why a decision was arrived at and what other issues have been addressed and discussed in terms of end of life care preferences is cause for concern if an individual moves across care boundaries and away from the care team with whom they had their initial discussions, something that is likely to happen in the approach to death.

For cases without evidence of end of life care discussions in the last four weeks of life, it was generally unclear as to whether discussions had either not taken place or were simply not recorded. It may be that patients were unable to engage in discussions at this time due to their mental and/or physical condition. However, this was not clear from the case notes. In a number of cases researchers encountered end of life care related discussions that were recorded *prior *to the last four weeks of life. These were not counted for the audit because they fell outside of the specified sampling window, and this is a potential limitation of the study design. However, it could also be argued that discussions should occur and be recorded at regular intervals as there is evidence from this and previous studies that patients' choices and concerns are not always clear cut and are likely to change over time, especially as death becomes imminent [15,20]. There needs to be a systematic and regular approach to reviewing any recorded wishes about end of life care already in place.

The findings presented above raise a number of concerns, not least because inconsistent or incomplete recording of patient expressed preferences means an individual's wishes may be unknown or ambiguous. If an individual subsequently loses capacity to decide for themselves, these wishes cannot reliably inform 'best interests' decisions [21]. In addition, poorly recorded or lack of recorded views and wishes may cause problems for patients when they move between services or into the care of different professionals within the same service. Patient choices and concerns may not be passed on or can be poorly communicated. This is highly undesirable given that where expressed preferences are clearly known they are more likely to be acted upon resulting in improved outcomes for both patients and family members [6]. The audit findings would also support this claim in that for the majority of cases with a recorded preference, the sampled services had enabled the patient to be in their preferred place of care (typically, their home/permanent residence).

It is recognised that service providers could easily be over-burdened with record-keeping and our group interview data clearly indicate that lack of time was already impacting on the nature of what was actually recorded about end of life care discussions in patients' notes. In addition, while end of life care tools appeared to be a useful prompt and support for staff initiating end of life care discussions, there was some evidence that they were regarded as a formal task to be routinely accomplished. This finding has been reported elsewhere [14,15] and may lead to feelings of pressure and obligation rather than leaving staff with the confidence to apply their personal discretion and judgement. However, more systematic approaches to recording end of life discussions are probably required in order to avoid the situation where information is simply retained in individual memories.

The results from our group interviews would suggest that participants recognised that records of patients^' ^last four weeks of care with respect to discussions relating to end of life care were limited and that improvements could be made. In particular, participants suggested that their record-keeping did not reveal the depth and quality of work actually undertaken, particularly in relation to conversations held with patients around preferred place of death and other aspects of end of life discussions. In addition, it was evident that there was some confusion about what it was necessary to record. If health care professionals were clearer about why information was needed and what purposes it served, it may help them in making decisions about how much detail to record and what issues to focus on.

### Study limitations

There are a number of limitations associated with this study. It is acknowledged that audit data reflects recording of discussions rather than actual discussions. We cannot assume that all patients were told about their condition or were aware of the nature of their situation or of the focus of the services they were receiving. Records may be incomplete or erroneous representations of services delivered. However, note-keeping may be revealing about perceived priorities, at least in terms of written communication and long-term documentation. Furthermore, the temporal specificity of the audit must be stressed. For the purposes of the audit, data collection was limited to care received in the last four weeks of life as we wanted to identify whether discussions were taking place at the very end of life. It may be, for example, that there were advanced discussions for some cases that were recorded prior to the last 4 weeks of life; these discussions would not be captured in the audit. In addition, due to limitations of time and resources, the researchers sampled from records that were kept by and within the services that they had access to; they did not obtain notes from all services with which a particular case had contact in the last four weeks of life.

## Conclusion

Overall the findings highlight that the formal recording and documentation of such discussions needs attention. If wishes and views are poorly recorded or communicated, or not recorded at all then they cannot inform care decisions once a patient can no longer express a choice or make decisions. We need to give further thought to how information may be adequately recorded, revised and updated and transferred across services to ensure that patients' preferences can be respected and realised wherever possible.

## Competing interests

The authors declare that they have no competing interests.

## Authors' contributions

KC and JS conceived the project and secured project funding. KC, JS, KA and NM contributed to the design of the study, development of the data collection tools and undertook the data collection. All authors contributed to data analysis and helped draft the manuscript. All authors have read and approved the final manuscript.

## Pre-publication history

The pre-publication history for this paper can be accessed here:

http://www.biomedcentral.com/1472-684X/10/18/prepub
